# Supplementary material for: Effective Hamiltonian-Based DNP Sequence Optimization
Source: J Phys Chem Lett. 2026 Mar 8;17(11):3084–90. doi: 10.1021/acs.jpclett.5c03855 (PMC13007014; doi:10.1021/acs.jpclett.5c03855)
Supplement: Supplementary file 1 [file jz5c03855_si_001.pdf]

Supporting Information: Effective Hamiltonian Based DNP Sequence Optimization

*Lorenzo Niccoli, Gian-Marco Camenisch, Matías Chávez and Matthias Ernst**

Institute for Molecular Physical Sciences, ETH Zürich, CH–8093 Zürich, Switzerland

Table of Contents

A. Pulsed DNP experiments **2**

Resonator Profile 2

Non–linearity of the Travelling Wave Tube (TWT) Amplifier 4

B. Pulsed DNP sequences **5**

Sequences with 72 pulses 5

Other pulsed DNP sequences 8

C. Effective Hamiltonian terms and transfer efficiency for the PLATO sequence **12**

D. DFT Calculation on the Trityl OX063 radical **13**

E. GAMMA Simulations **13**

F. Excitation Pulse Profile **13**

G. Droop Measurements **14**

H. DNP Build-up curves **16**

References **17**

A. Pulsed DNP experiments

In this section we report the resonator profile and the measure of the non–linearity of the travelling wave tube (TWT) amplifier of the spectrometer used to characterize all the pulsed DNP sequences reported in the main text (Fig. S1-S2). The experimental protocols used for these measurements are analogous to the ones described in Camenisch et al[1].

Resonator Profile

The experiment to record a resonator profile is a three-pulse experiment. The first pulse serves as a nutation pulse and is incremented from 0 to 128 ns in steps of 2 ns at maximum power (digital amplitude = 1). Electron spin magnetization after a delay T~$5T_{2,e}$ is observed with a two-pulse Hahn echo experiment. The experiment is measured for different electron offsets with respect to the center of the resonator. The external magnetic field is swept to be on resonant with the mw frequency. The resulting resonator profile indicates the largest B_1field (or strongest Rabi frequency) that can be obtained at a certain electron offset.

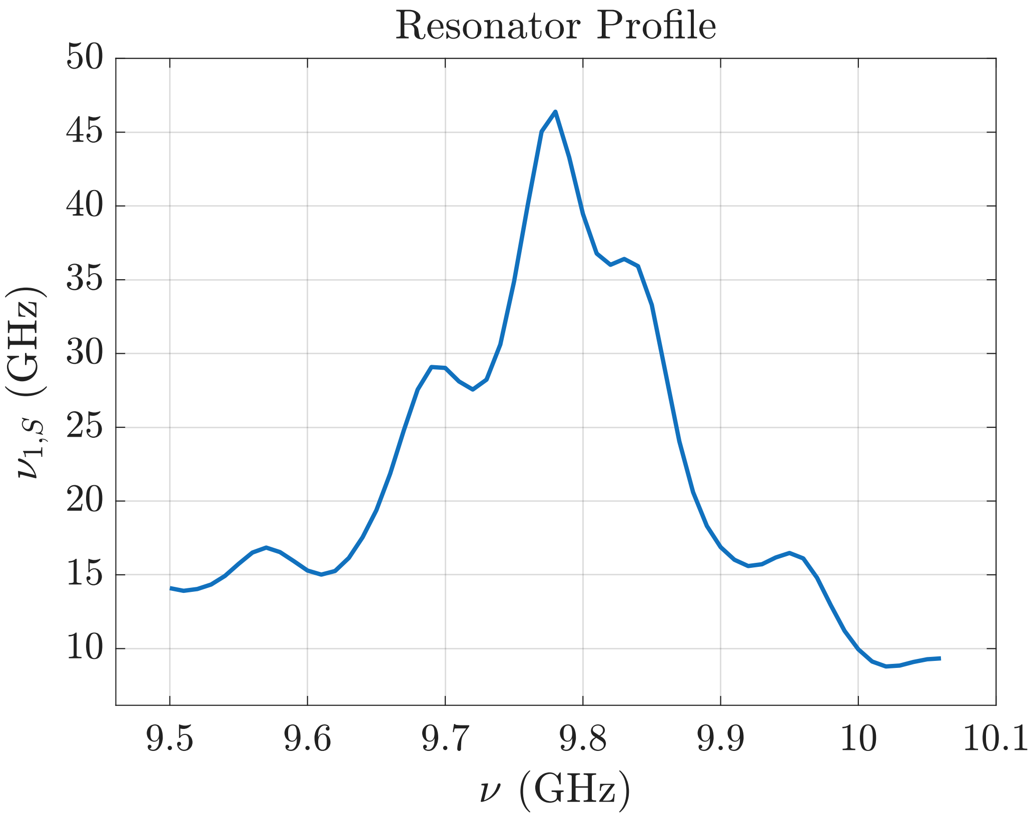

Figure S1 Resonator profile for the trityl radical. This profile indicates the largest B_1 field that can be obtained at a certain offset. The center of the resonator is at ν = 9.78 GHz.

Non–linearity of the Travelling Wave Tube (TWT) Amplifier

The experiment to record the non-linearity of the TWT amplifier is a three-pulse experiment. The first pulse serves as a nutation pulse and is incremented from 0 to 512 ns in steps of 2 ns at various digital amplitude. Electron spin magnetization after a delay a delay T~$5T_{2,e}$ is then observed with a two-pulse Hahn echo experiment. The experiment is measured at the center of the resonator ~ 9.78 GHz. Fitting for both the dependence of Rabi frequency on digital amplitude and the dependence of digital amplitude on required Rabi frequency of the TWT non-linearity curve by polynomials of fourth order allows the mapping between the digital amplitude and the Rabi frequency. The TWT non-linearity curve together with the resonator profile is used to compensate the limited width of the microwave resonator mode and differences in non-linearity of the TWT during the acquisition of a DNP profile.

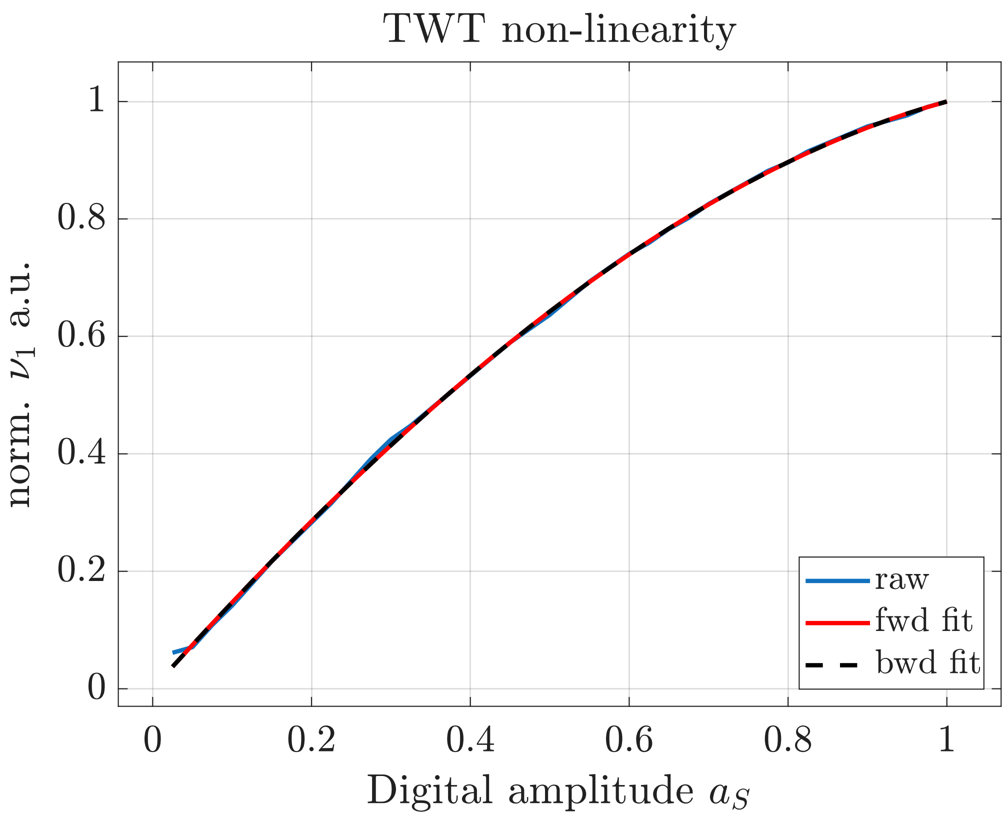

Figure S2 Non-linearity of the TWT amplifier for the trityl radical. The TWT non-linearity curve together with the resonator profile is used to compensate the limited width of the microwave resonator mode and differences in non-linearity of the TWT during the acquisition of a DNP profile.

B. Pulsed DNP sequences

Sequences with 72 pulses

In this section we report the relative amplitude of all the optimized sequences discussed in the main text. The offset for the off-resonance sequence is +50 MHz. A graphical representation of each pulse sequence in shown in Fig. S3.

On-resonance DNP sequence

Bandwidth = 100 MHz, microwave power = 25 MHz

[0.2799 0.9119 1.0000 1.0000 –0.5883 –1.0000 0.0340 –1.0000 –1.0000 –1.0000 –1.0000 1.0000 1.0000 –0.2218 –1.0000 –1.0000 –1.0000 –0.6442 1.0000 0.7882 –0.1632 1.0000 1.0000 0.8996 –1.0000 0.0042 –0.9758 –1.0000 –1.0000 –1.0000 –1.0000 1.0000 1.0000 0.2030 –1.0000 0.0389 –1.0000 –1.0000 –0.5097 1.0000 1.0000 0.9171 –0.6976 –0.5858 1.0000 1.0000 1.0000 0.6177 –1.0000 0.7784 1.0000 0.1306 0.9129 1.0000 –1.0000 –1.0000 –1.0000 –0.9784 –0.5475 0.5806 1.0000 1.0000 –0.1561 –0.7739 1.0000 1.0000 1.0000 1.0000 –0.5312 –1.000 0.9177 1.0000]

Bandwidth = 80 MHz, microwave power = 25 MHz

[–1.0000 –1.0000 –1.0000 0.8048 1.0000 0.6833 0.8304 1.0000 0.2942 –1.0000 –1.0000 –0.4535 –1.0000 –1.0000 –1.0000 –1.0000 1.0000 1.0000 0.5833 –1.0000 –0.8229 –0.7501 –1.0000 –1.0000 1.0000 1.0000 1.0000 –0.4981 –1.0000 1.0000 1.0000 1.0000 0.9914 –1.0000 0.5821 1.0000 0.4121 1.0000 1.0000 –1.0000 –1.0000 –1.0000 –0.8430 –0.5235 0.0848 1.0000 1.0000 0.7286 –1.0000 –0.5017 1.0000 1.0000 1.0000 1.0000 –1.0000 –0.1049 1.0000 1.0000 1.0000 1.0000 1.0000 –1.0000 –1.0000 –0.9327 –0.1575 –0.8397 –0.5034 –1.0000 0.5989 1.0000 1.0000 –1.0000]

Off-resonance DNP sequence

Bandwidth = 20 MHz, microwave power = 20 MHz

[1.0000 –0.8886 –1.0000 1.0000 0.7763 –1.0000 –1.0000 1.0000 0.5991 –0.2579 1.0000 1.0000 1.0000 –1.0000 –1.0000 0.5101 1.0000 0.0544 0.3526 –0.3569 –1.0000 0.4502 1.0000 –1.0000 –1.0000 1.0000 1.0000 –1.0000 –1.0000 1.0000 1.0000 –1.0000 0.6908 –1.0000 –1.0000 1.0000 1.0000 –1.0000 0.3166 0.2955 –1.0000 –1.0000 1.0000 1.0000 –1.0000 –1.0000 1.0000 1.0000 1.0000 –1.0000 1.0000 1.0000 –1.0000 –1.0000 1.0000 –1.0000 –1.0000 1.0000 –0.4263 –1.0000 –0.0379 1.0000 1.0000 –1.0000 –1.0000 1.0000 1.0000 –0.8975 0.8810 –1.0000 –1.0000 1.0000]

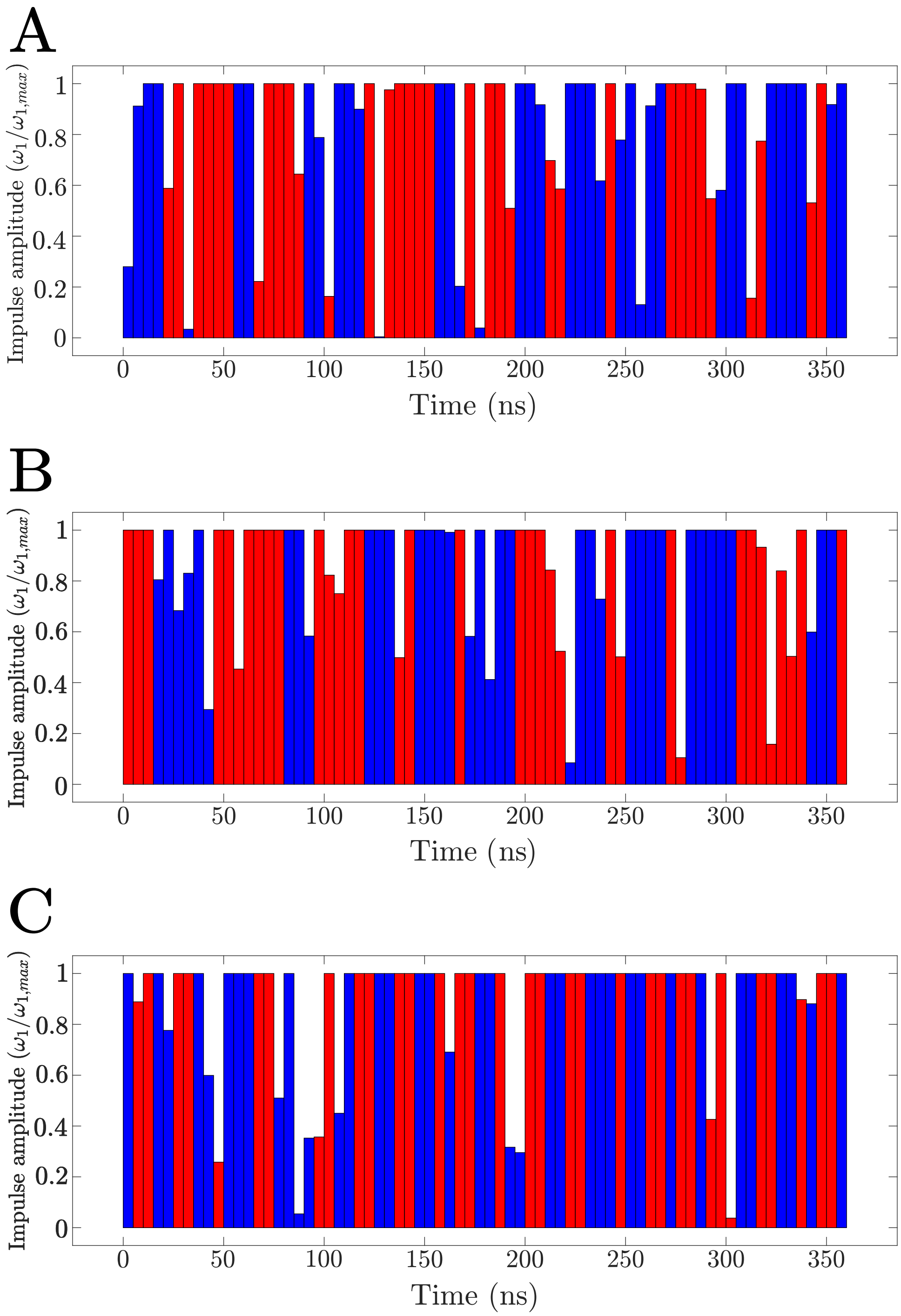

Figure S3. Pulse sequence diagrams: (A) On-resonance, 100 MHz bandwidth, 25 MHz amplitude; (B) On-resonance, 80-MHz bandwidth, 25 MHz amplitude; (C) Off-resonance (50 MHz center), 20 MHz bandwidth, 20 MHz amplitude. Blue and red denote positive and negative amplitudes.

Other pulsed DNP sequences

In this section, we report additional optimized DNP sequences that are not discussed in the main text. These include both on-resonance and off-resonance sequences consisting of 48 or 96 pulses each with 5 ns length, covering electron offset bandwidths of 80 MHz or 120 MHz for the on-resonance case, and bandwidths of 20 MHz or 40 MHz centered at 40 or 50 MHz for the off-resonance case. The parameters for all optimized sequences are summarized in Table S1, and the sequences are labeled A–F for convenience. The corresponding experimental data, acquired as described in the main text, are compared with effective Hamiltonian calculations and GAMMA simulations in Figure S4. The repetition number was set equal to 4 for the sequences with 48 pulses and to 2 for the sequences with 96 pulses. The relative amplitudes of all these sequences are reported later in this section.

Table S1. Sequence type, number of pulses, microwave power and target bandwidth for the DNP sequences (A-F) presented in this section.

**Label**	**Sequence type**	**N° pulses**	**Microwave Power (MHz)**	**Bandwidth (MHz)**
A	On-resonance	48	25	80
B	On-resonance	96	25	120
C	On-resonance	48	30	80
D	On-resonance	96	30	120
E	Off-resonance	48	15	20 (center = 40 MHz)
F	Off-resonance	96	15	40 (center = -50 MHz)

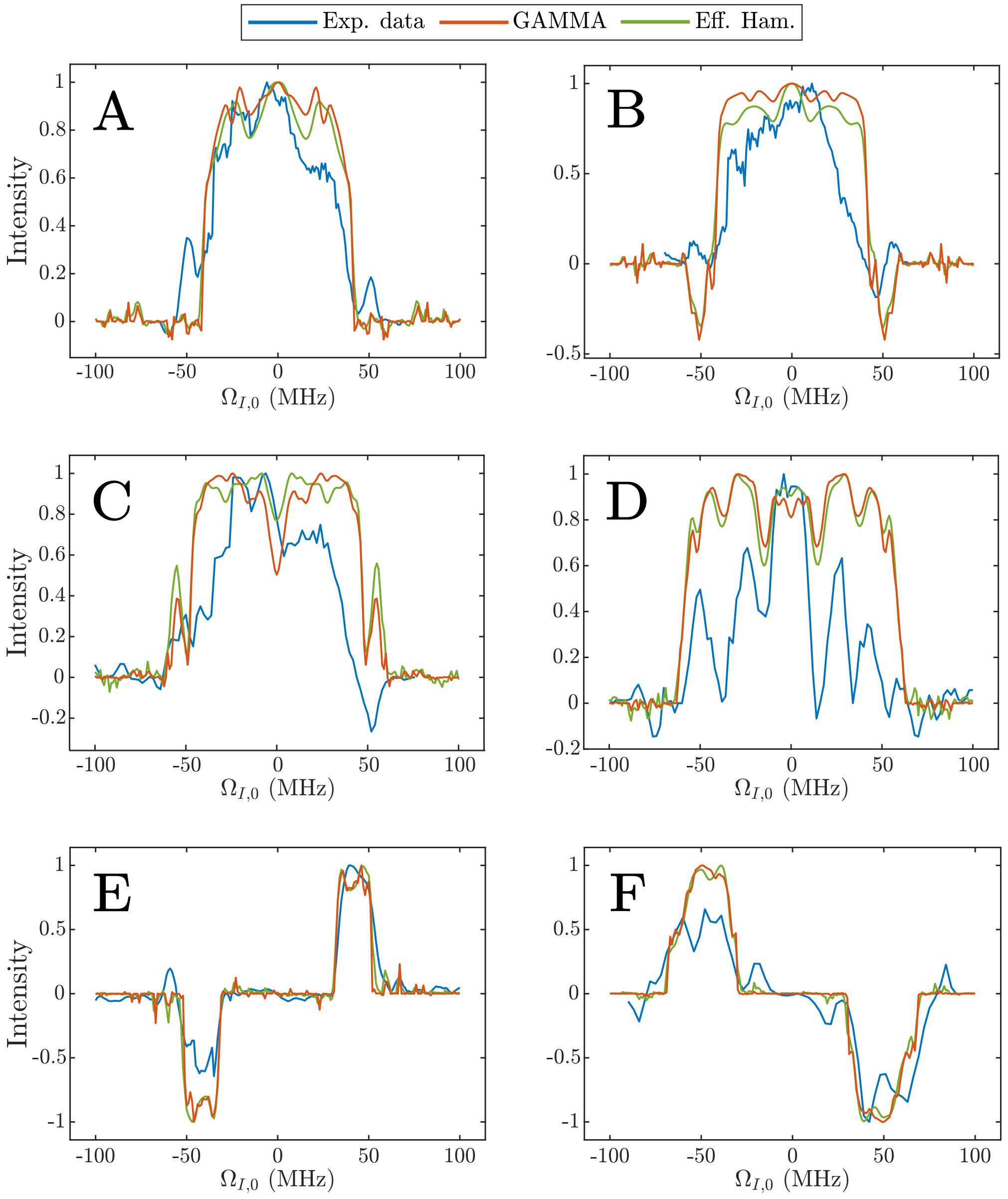

Figure S4. Comparison of experimental data with effective Hamiltonian and GAMMA calculations for different optimized pulse sequences. Sequences A and C were acquired with 25 MHz microwave power, B and D with 30 MHz, and E and F with 15 MHz. On-resonance sequences: (A) 48 pulses, bandwidth: 80 MHz; (B) 96 pulses, bandwidth: 120 MHz; (C) 48 pulses, bandwidth: 80 MHz; (D) 96 pulses, bandwidth: 120 MHz. Off-resonance sequences: (E) 48 pulses, bandwidth: 20 MHz centered at 40 MHz; (F) 96 pulses, bandwidth: 40 MHz centered at 50 MHz. Each profile has been normalized to their respective maximum value to allow the comparison.

Sequence A

N° Pulses = 48, Bandwidth = 80 MHz, Microwave Power = 25 MHz

[-0.2888 -1.0000 0.6968 1.0000 1.0000 1.0000 -0.6580 0.9052 0.5621 1.0000 1.0000 -1.0000 -1.0000 -1.0000 -1.0000 -0.3123 -0.0464 1.0000 1.0000 0.7573 -1.0000 -0.6967 1.0000 1.0000 1.0000 1.0000 -1.0000 0.5312 1.0000 1.0000 0.7598 1.0000 1.0000 -1.0000 -1.0000 -1.0000 0.3287 -1.0000 -1.0000 -0.3524 1.0000 1.0000 0.9471 -1.0000 -1.0000 -1.0000 -1.0000 0.3741**]**

Sequence B

N° Pulses = 96, Bandwidth = 120 MHz, Microwave Power = 25 MHz

[-0.9997 1.0000 1.0000 1.0000 -0.4583 0.9999 -0.5605 -0.9999 1.0000 1.0000 1.0000 0.4129 -0.9985 0.5609 0.9999 0.6287 -1.0000 -1.0000 -0.7696 -0.3387 -0.5094 1.0000 1.0000 -0.3268 -1.0000 -1.0000 -1.0000 -0.1542 -1.0000 -1.0000 -1.0000 1.0000 1.0000 -0.4811 0.5559 1.0000 0.5627 -1.0000 -1.0000 -1.0000 -0.2017 1.0000 0.2772 -1.0000 -1.0000 -0.9717 0.7483 -1.0000 -1.0000 1.0000 1.0000 1.0000 -0.0913 1.0000 -0.2923 -1.0000 1.0000 1.0000 1.0000 0.3393 -0.9999 0.3198 1.0000 0.2831 -1.0000 -1.0000 -0.3647 -0.7959 0.1266 1.0000 1.0000 -0.4888 -1.0000 -1.0000 -1.0000 -0.0839 -1.0000 -1.0000 -0.7032 1.0000 0.9720 -0.5770 1.0000 1.0000 0.0723 -0.8969 -1.0000 -1.0000 0.1382 1.0000 -0.0281 -0.9884 -0.7306 -0.9999 0.3825 -0.3546]

Sequence C

N° Pulses = 48, Bandwidth = 80 MHz, Microwave Power = 30 MHz

[0.0536 -1.0000 -1.0000 -1.0000 -0.8482 0.8101 1.0000 0.9027 -0.6312 -0.2786 -0.9251 0.0870 -0.8777 -1.0000 -1.0000 1.0000 1.0000 0.4270 1.0000 1.0000 -0.0302 -1.0000 1.0000 1.0000 1.0000 1.0000 -0.7618 -0.3275 0.8531 0.9239 0.3897 -0.5858 -0.8759 -1.0000 -1.0000 -1.0000 1.0000 1.0000 0.5500 0.4936 0.0071 -1.0000 1.0000 1.0000 1.0000 0.4190 -1.0000 1.0000**]**

Sequence D

N° Pulses = 96, Bandwidth = 120 MHz, Microwave Power = 30 MHz

[-0.3448 -1.0000 -0.4251 1.0000 1.0000 1.0000 1.0000 -1.0000 -1.0000 0.9999 0.5869 -0.3181 1.0000 1.0000 1.0000 -0.6460 0.0252 0.4508 0.1485 0.4072 -1.0000 -1.0000 -0.9846 1.0000 -0.1442 0.7468 1.0000 1.0000 -1.0000 -0.0317 1.0000 1.0000 1.0000 -0.8755 -1.0000 0.8315 1.0000 -0.1497 1.0000 1.0000 0.7510 -1.0000 -0.6639 0.4474 -1.0000 -1.0000 -1.0000 0.9999 0.5893 -1.0000 -1.0000 -0.9999 -1.0000 0.6244 1.0000 -0.3509 -0.1631 1.0000 1.0000 -0.9200 -0.6451 -0.9996 -0.1525 -0.6436 -0.9999 -1.0000 0.7728 1.0000 0.8722 -1.0000 -0.1966 -0.1675 -1.0000 -1.0000 1.0000 1.0000 0.2215 1.0000 0.9999 0.2195 0.8148 -1.0000 -1.0000 0.4682 0.6243 1.0000 1.0000 1.0000 -0.6603 -0.4285 0.9680 1.0000 -0.4778 -1.0000 -1.0000 0.9999]

Sequence E

N° Pulses = 48, Bandwidth = 20 MHz (centre = 40 MHz), Microwave Power = 15 MHz

[-1.0000 1.0000 1.0000 1.0000 1.0000 -1.0000 -1.0000 -1.0000 1.0000 1.0000 -1.0000 -1.0000 0.3870 1.0000 1.0000 -1.0000 -1.0000 1.0000 -1.0000 -1.0000 1.0000 1.0000 -1.0000 -0.7340 1.0000 1.0000 1.0000 -1.0000 -1.0000 1.0000 1.0000 1.0000 -0.0950 -1.0000 -1.0000 1.0000 1.0000 -1.0000 -1.0000 1.0000 1.0000 -0.8733 -1.0000 0.8681 0.7675 0.8575 -1.0000 -1.0000 **]**

Sequence F

N° Pulses = 96, Bandwidth = 40 MHz (centre = -50 MHz), Microwave Power = 15 MHz

[-1.0000 1.0000 -0.4483 -1.0000 0.7674 0.7882 -1.0000 -0.7308 0.3295 1.0000 0.8511 -1.0000 -1.0000 0.6118 1.0000 -0.1929 1.0000 1.0000 -1.0000 -1.0000 1.0000 -0.1193 -0.1778 1.0000 -1.0000 -1.0000 1.0000 1.0000 -0.8296 -1.0000 1.0000 1.0000 -1.0000 -1.0000 1.0000 -0.0648 1.0000 1.0000 -1.0000 -1.0000 1.0000 0.6921 0.2611 -0.6332 -1.0000 1.0000 0.8796 -1.0000 -0.9336 1.0000 -0.8427 -0.7389 1.0000 1.0000 0.0141 -1.0000 -0.2760 1.0000 1.0000 -1.0000 -1.0000 -1.0000 0.1884 -0.6529 1.0000 1.0000 -1.0000 -1.0000 0.7279 -1.0000 -0.5143 1.0000 -1.0000 -1.0000 1.0000 1.0000 -1.0000 -0.9277 1.0000 1.0000 -1.0000 -1.0000 0.4094 1.0000 0.3373 1.0000 -1.0000 -1.0000 1.0000 1.0000 -0.5328 1.0000 -1.0000 1.0000 1.0000 -1.0000]

C. Effective Hamiltonian terms and transfer efficiency for the PLATO sequence

In Figure S5 we report the calculation for the PLATO sequence[2] of the effective Hamiltonian terms as a function of the electron frequency offset, together with the corresponding transfer efficiency evaluated as a function of the normalized microwave amplitude (32 MHz) and the electron frequency offset $\Delta\omega_S/2\pi$. As in the main text, all effective Hamiltonian terms are normalized to the maximum value of the ZQ term.

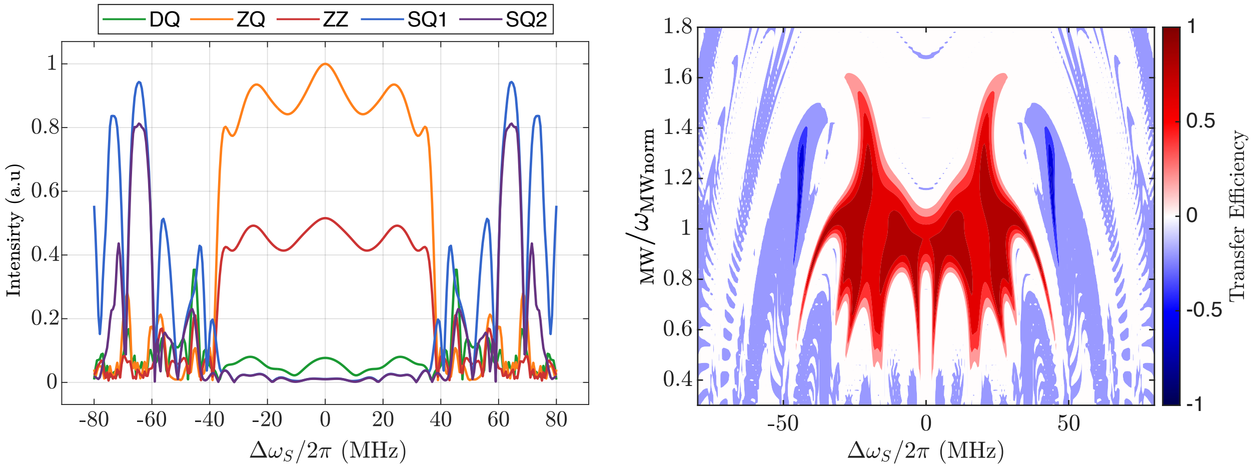

Figure S5 (Left) Effective Hamiltonian terms (DQ, ZQ, ZZ, SQ1, SQ2) in function of the electron frequency offset. (Right) Transfer efficiency as a function of the electron frequency offset and the microwave amplitude.

D. DFT Calculation on the Trityl OX063 radical

The DFT calculation on Trityl OX063 have been performed with ORCA 5[3,4]. The initial geometry was generated with Avogadro[5] and optimized using the B3LYP[6,7] functional with D3BJ[8,9] dispersion corrections, the def2-SVP basis set, and TightSCF options.

Hyperfine couplings were then computed on the optimized geometry using the B3LYP functional, the EPR-III basis set[10], and the D3BJ and TightSCF options. For sulfur atoms was set the def2-TZVPPD[11,12] basis set.

The most intense hyperfine coupling (2.64 MHz) was used as the reference value for the GAMMA[13] calculations presented in the main text.

E. GAMMA Simulations

The GAMMA[13] simulations were performed using a two-spin electron-proton system with an electron-proton distance of $r_{eH} \approx 4\text{Å}$. And an axial g-tensor with a slightly g-anisotropy $g_{xx} = g_{yy} = 2.00319$ and $g_{zz} = 2.00258$. The values for the g-tensor are reported in Ref.[14]. For the relative HFI tensor the Euler angles obtained from the DFT calculations were used $(\alpha, \beta, \gamma) = (142.5°, 303.9°, 236.7°)$. Relaxation was modeled in Liouville space by using the random-field approach.

F. Excitation Pulse Profile

In Figure S6 we show the theoretical excitation pulse profile for a 90° pulse with a length of 6 ns and a phase +y. Fit of the field-swept EPR spectrum at X-band (around 0.35 T) to a Gaussian line shape gave a FWHM of 6.3 MHz. Numerical simulations of the components of the angular spin operator $\hat{\vec{S}}$ are shown in green for $\hat{S}_x$, blue for $\hat{S}_y$ and black for $\hat{S}_z$. One can clearly see that over the spectral region of the trityl the 90° pulse has almost a perfect uniform excitation.

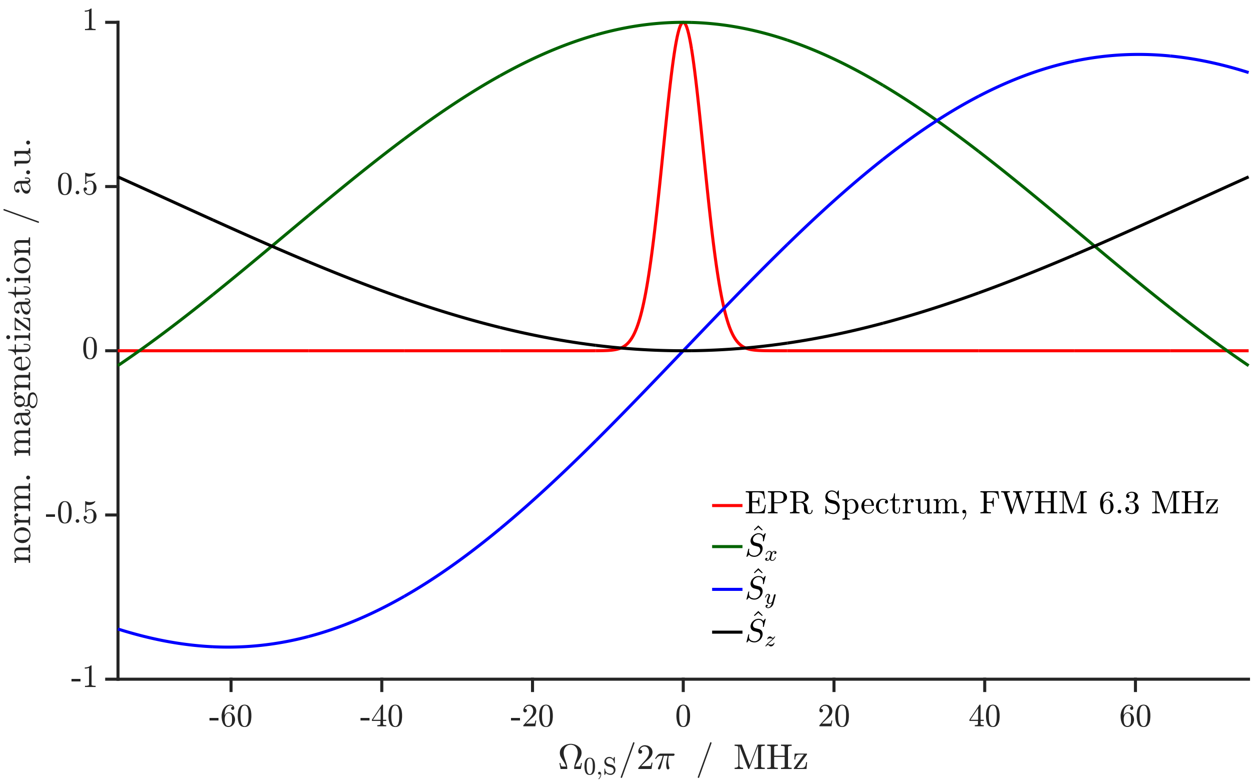

Figure S6 Theoretical excitation pulse profile for a 90° pulse with a length of 6 ns and a phase +y for an EPR spectrum with a FWHM of 6.3 MHz.

G. Droop Measurements

The mw signal amplified by the TWT was passing a first 3-port directional coupler (Narda 4015C-30, frequency range 7-12.4 GHz). Almost all of the mw signal was directed towards the resonator. -30 dB of the initial mw signal was transmitted through a second 3-port directional coupler of the same type for further attenuation of the mw irradiation before passing it through a diode (DD-20-218-5PF-3-P-M, frequency range 2-18 GHz, rise time about 5 ns). The attenuated mw signal was then measured with an oscilloscope (Rhode Schwarz RTA 4004).

Droop measurements for 100%, 75% and 50% amplitude are shown in Fig. S7. We can see from Fig. S7 that the amplitude of the mw drops significantly over time.

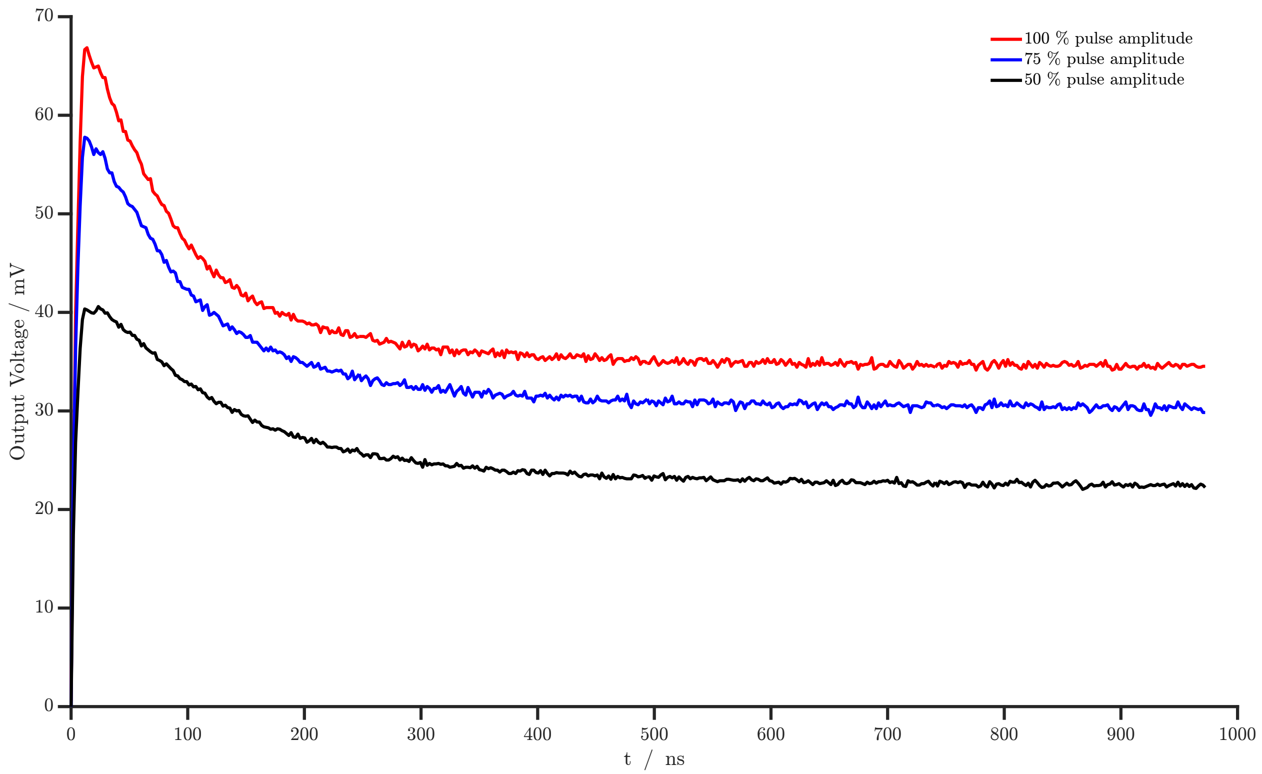

Figure S7. Measurement of the droop for 100% pulse amplitude (red), 75% pulse amplitude (blue) and 50% pulse amplitude (black).

H. DNP Build-up curves

In this section we report the build-up curves of the PLATO sequence and the optimized sequence shown in Figure 3 in the main text. Each point of the build-up curve has been acquired as described in the main text. Notably the enhancement value at the steady-state reported for the PLATO sequence are lower than what reported previously[2]. We attribute this difference to the significant microwave droop discussed in section G of the SI.

The build-up times for the PLATO sequence is 5.7 s and for the optimized sequence is 6.0 s.

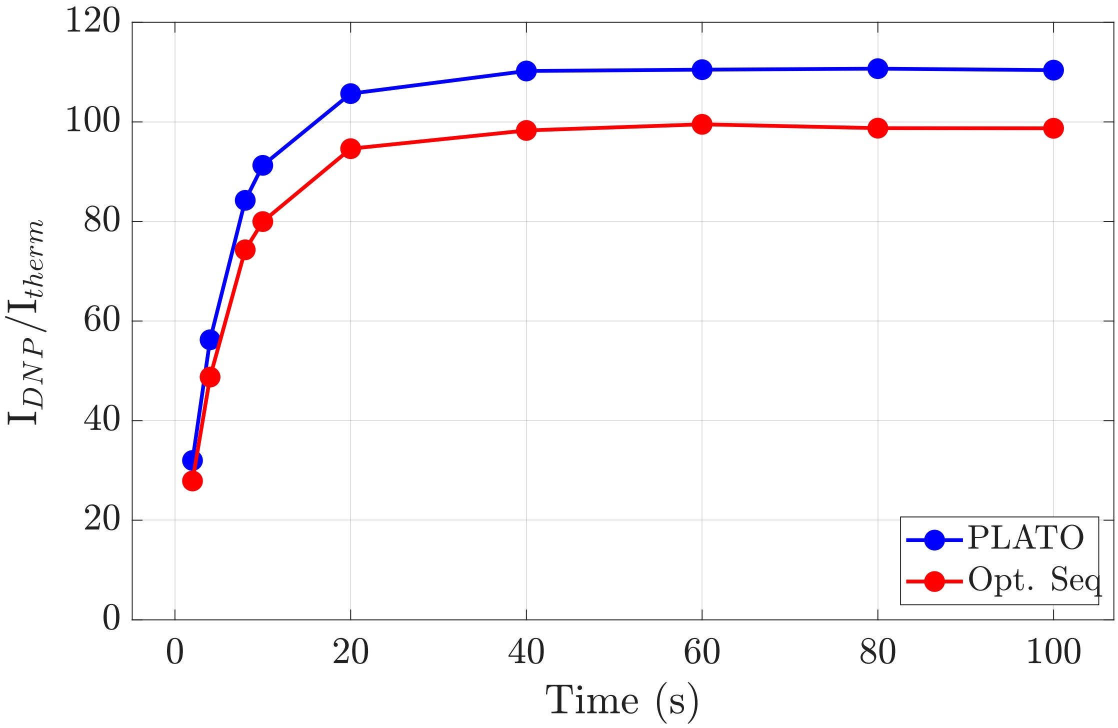

Figure S8. Build-up curves for the PLATO sequence and for the optimized pulsed sequence shown in Figure 3 of the main text.

References

(1) Camenisch, G.-M.; Wili, N.; Jeschke, G.; Ernst, M. Pulsed Dynamic Nuclear Polarization: A Comprehensive Floquet Description. *Phys. Chem. Chem. Phys.* **2024**, *26* (25), 17666–17683. DOI: 10.1039/D4CP01788A.

(2) Nielsen, A. B.; Carvalho, J. P. A.; Goodwin, D. L.; Wili, N.; Nielsen, N. C. Dynamic Nuclear Polarization Pulse Sequence Engineering Using Single-Spin Vector Effective Hamiltonians. *Phys. Chem. Chem. Phys.* **2024**, *26* (44), 28208–28219. DOI: 10.1039/D4CP03041A.

(3) Neese, F. The ORCA Program System. *WIREs Comput. Mol. Sci.* **2012**, *2* (1), 73–78. DOI: 10.1002/wcms.81.

(4) Neese, F. Software Update: The ORCA Program System—Version 5.0. *WIREs Comput. Mol. Sci.* **2022**, *12* (5), e1606. DOI: 10.1002/wcms.1606.

(5) Hanwell, M. D.; Curtis, D. E.; Lonie, D. C.; Vandermeersch, T.; Zurek, E.; Hutchison, G. R. Avogadro: An Advanced Semantic Chemical Editor, Visualization, and Analysis Platform. *J. Cheminform* **2012**, *4* (1), 17. DOI: 10.1186/1758-2946-4-17.

(6) Becke, A. D. Density-functional Thermochemistry. III. The Role of Exact Exchange. *J Chem. Phys.* **1993**, *98* (7), 5648–5652. DOI: 10.1063/1.464913.

(7) Stephens, P. J.; Devlin, F. J.; Chabalowski, C. F.; Frisch, M. J. Ab Initio Calculation of Vibrational Absorption and Circular Dichroism Spectra Using Density Functional Force Fields. *J. Phys. Chem.* **1994**, *98* (45), 11623–11627. DOI: 10.1021/j100096a001.

(8) Grimme, S.; Antony, J.; Ehrlich, S.; Krieg, H. A Consistent and Accurate Ab Initio Parametrization of Density Functional Dispersion Correction (DFT-D) for the 94 Elements H-Pu. *J. Phys. Chem.* **2010**, *132* (15), 154104. DOI: 10.1063/1.3382344.

(9) Grimme, S.; Ehrlich, S.; Goerigk, L. Effect of the Damping Function in Dispersion Corrected Density Functional Theory. *J. Comput. Chem.* **2011**, *32* (7), 1456–1465. DOI: 10.1002/JCC.21759.

(10) Rega, N.; Cossi, M.; Barone, V. Development and Validation of Reliable Quantum Mechanical Approaches for the Study of Free Radicals in Solution. *J. Chem. Phys.* **1996**, *105* (24), 11060–11067. DOI: 10.1063/1.472906.

(11) Weigend, F.; Ahlrichs, R. Balanced Basis Sets of Split Valence, Triple Zeta Valence and Quadruple Zeta Valence Quality for H to Rn: Design and Assessment of Accuracy. *Phys. Chem. Chem. Phys.* **2005**, *7* (18), 3297–3305. DOI: 10.1039/B508541A.

(12) Rappoport, D.; Furche, F. Property-Optimized Gaussian Basis Sets for Molecular Response Calculations. *J. Chem. Phys.* **2010**, *133* (13), 134105. DOI: 10.1063/1.3484283.

(13) Smith, S. A.; Levante, T. O.; Meier, B. H.; Ernst, R. R. Computer Simulations in Magnetic Resonance. An Object-Oriented Programming Approach. *J. Magn. Reson.* **1994**, *106* (1), 75–105. DOI: 10.1006/jmra.1994.1008.

(14) Lumata, L., Kovacs, Z., Sherry, A. D., Malloy, C., Hill, S., Van Tol, J., Yu, L., Song, L., Merritt, M. E. Electron spin resonance studies of trityl OX063 at a concentration optimal for DNP. *Phys. Chem. Chem. Phys*. **2013,** *15*, 9800-9807. DOI:10.1039/c3cp50186h.
