# Supplementary material for: Effective Hamiltonian-Based DNP Sequence Optimization
Source: J Phys Chem Lett. 2026 Mar 8;17(11):3084–90. doi: 10.1021/acs.jpclett.5c03855 (PMC13007014; doi:10.1021/acs.jpclett.5c03855)
Supplement: Supplementary file 2 [file jz5c03855_si_002.pdf]

jz-2025-03855p.R1

Name: Peer Review Information for "Effective Hamiltonian Based DNP Sequence Optimization"

First Round of Reviewer Comments

Reviewer: 1

Comments to the Author

This manuscript presents an optimization framework for pulsed dynamic nuclear polarization (DNP) sequences based on continuous Floquet theory and first-order effective Hamiltonians. The authors design both on-resonance and off-resonance sequences, validate them experimentally at X-band, and benchmark their performance against the PLATO sequence. The work combines theoretical modeling, numerical simulations, and experimental measurements in a coherent manner. In my opinion, the study is suitable for publication in JPCL, but several methodological aspects require clarification, and several cosmetic issues should be addressed before acceptance. I recommend minor revision.

Specific Comments Page 3/21, line 10:

Is reference 32 correctly cited here? It does not appear to be directly relevant to the MIRROR or symmetry-based C/R sequences mentioned in the text.

General comment on methodology and reproducibility:

The manuscript contains many technical terms and algorithmic references (e.g., fmincon, rng, twister), as well as script-specific parameters such as units, amplitude ranges, limits, and time steps.

To improve transparency and reproducibility, I strongly encourage the authors to deposit the MATLAB (or equivalent) scripts in an online repository. This would greatly help readers understand the optimization workflow and reproduce the results.

Page 5/21, line 6:

What is the difference between SQ1 and SQ2? A brief explanation would help readers unfamiliar with this notation.

Page 5/21, line 42:

What does “ZZ” refer to? This term is not defined in the text and should be clarified.

Page 7/21, line 10:

If n = 3, what is the corresponding mixing time? I assume it must be on the order of a few hundred nanoseconds (< 1 μs) before T_2e relaxation becomes significant. Later in the manuscript, the authors mention that 72 pulses × 5 ns = 360 ns were used. Does this imply that n = 1 corresponds to 24 pulses?

Page 9/21, line 9:

The reported enhancement factors of 23 and 70 appear low compared to values in other pulsed DNP studies, which often exceed 300 under optimized or saturated conditions. Is this primarily due to the relatively short 2-s DNP build-up time used here? Would the authors expect enhancements above 200 if longer build-up times (>10 s) were employed?

Figure-Related Comments Figure 1A:

Since the entire subfigure (A) is located on the left, the word “left” is redundant. The caption could be simplified by changing “top left” to “top” and “middle left” to “middle.”
Figure 2:

Are subfigures (A) and (D) slices extracted from the 2D plots in (B) and (E) at a microwave amplitude of 1 (i.e., along the y-axis)? Clarification would help readers interpret the figure.

Figure 4:

The labels (A/B/C) are missing from the figure panels.

There is also a typographical error in the caption (“centered at 50…”).

Figure 4C:

Regarding the mismatch between experiment and simulation/theory: the simulations use a two-spin model. Could multispin effects be relevant, especially at large offsets? For example, two-nuclear-spin contributions may become significant (see DOI: 10.1126/sciadv.aax2743).

Additional Methodological Clarification GAMMA simulations:

The manuscript would benefit from a more detailed description of the spin parameters used in the GAMMA simulations. Specifically, please provide the values of T_1n, T_2n, T_1e, T_2e, and the hyperfine tensor employed.

Reviewer: 2

Comments to the Author

This manuscript describes the theoretical analysis of pulse DNP sequences (on-resonant and off-resonant) by continuous Floquet theory. This is an extension of an earlier work of this research group (PCCP 2024) using operator-based and matrix-based discrete Floquet theory. The authors use and demonstrate their approach by optimizing an on-resonant and off-resonant pulse sequence with amplitude modulated MW pulses with regard to the zero-quantum operator between electron and nuclear spin. This is interesting, since pulse DNP sequences have a large potential to increase DNP efficiency for polarizing agents with broad EPR spectra and potentially also at high magnetic fields (if the necessary pulse and frequency modulated MW power becomes technically available in the future).

My main points of critic with the existing manuscript are:

The authors claim that their approach is superior to already existing approaches (as Single-Spin Vector effective Hamiltonian Theory) because it can better treat near-resonance conditions. This is important because this conditions will always appear with the limited MW power available. Unfortunately, I do not see in the paper a demonstration of this superior behavior. The authors only show comparison with numerical GAMMA spin dynamic simulations, which demonstrate that the approach is valid, but does not demonstrate that it is superior to SSV-EHT.

Secondly, the authors have used their approach to optimize an on-resonance MW pulse and compare it with the published PLATO sequence (optimized with SSV-EHT). Their analysis of the results are somewhat confusing. They state that their new developed MW pulse is superior because it achieves the same bandwidth with somewhat reduced MW power (20%). Later-on, they state that the enhancement of their pulse sequence is 20% lower! It is not clear to me, why they did not compare with the same maximum MW power (which was available for their PLATO control experiment) and why they are not more precise in this comparison. By the way, the reader has to guess in Figure 3A which trace belongs to the new sequence and which one to the PLATO sequence; this has to be explained!

I would propose, that the authors make these two points more clear in a revised version of the manuscript before publication!

Minor points:

Despite it was already done like that in earlier publications I would favor to have the pulse amplitudes shown from +1 to -1 in one color instead of showing only the magnitude of the B1 field with two colors.

Please give the pulse length of the MW rectangular pi/2 pulse

The authors describe that for on-resonant cases they start from the Sx vector. On the other hand, with the given MW field strength, this is not exactly correct. For off-resonant spins the starting vector will deviate. Anyway, to me it is unclear, why this first pulse is not included in the whole calculation.

The authors write about tolerance of their sequence against MW field strength inhomogeneity, but I did not find how this was extracted and how such profiles were taken into account.

Please also specify the ZZ Term (Spin-alignment) in the manuscript

The authors write about power droop during the pulse. How was this evaluated?

How was the TWT non-linearity at high MW powers taken into account? Is there also a MW phase shift in the non-linear regime?

Author's Response to Peer Review Comments:

In the following response letter, the reviewer’s comments are shown in black, and responses from the authors are shown in red.

Reviewer: 1

Recommendation: This paper is publishable subject to minor revisions noted. Further review is not needed.

Comments:

This manuscript presents an optimization framework for pulsed dynamic nuclear polarization (DNP) sequences based on continuous Floquet theory and first-order effective Hamiltonians. The authors design both on-resonance and off-resonance sequences, validate them experimentally at X-band, and benchmark their performance against the PLATO sequence. The work combines theoretical modeling, numerical simulations, and experimental measurements in a coherent manner. In my opinion, the study is suitable for publication in JPCL, but several methodological aspects require clarification, and several cosmetic issues should be addressed before acceptance. I recommend minor revision.

Specific Comments

Page 3/21, line 10: Is reference 32 correctly cited here? It does not appear to be directly relevant to the MIRROR or symmetry-based C/R sequences mentioned in the text.

Thank you for spotting this mistake. This is obviously a wrong citation. We added the correct citation in the main text (https://doi.org/10.1016/j.ssnmr.2022.101834) **General comment on methodology and reproducibility**:

The manuscript contains many technical terms and algorithmic references (e.g., fmincon, rng, twister), as well as script-specific parameters such as units, amplitude ranges, limits, and time steps. To improve transparency and reproducibility, I strongly encourage the authors to deposit the MATLAB (or equivalent) scripts in an online repository. This would greatly help readers understand the optimization workflow and reproduce the results.

We have reduced the number of technical terms since we do not describe the optimization procedure in detail by removing the sentence " The generation of the starting sequences was performed using the *rng* MATLAB function, with the *twister* algorithm”. We believe that all the other terms are standard spectroscopic terms that do not need any explanation or should confuse readers. We have prepared an online repository with the experimental and simulation results. In the longer run, we will also make the implementation publicly available but we are not yet at a point where we believe that this makes sense. The scripts need to be made more general and optimized and documented in a better way. This will

happen after we publish a more methodological paper about the optimization procedures used here.

Page 5/21, line 6: What is the difference between SQ1 and SQ2? A brief explanation would help readers unfamiliar with this notation.

Page 5/21, line 42: What does "ZZ" refer to? This term is not defined in the text and should be clarified.

On page 3/4 we have changed the text to: " The optimization process involves minimizing the difference between the coefficients of the zero-quantum (ZQ) and double-quantum (DQ), twospin longitudinal order (ZZ), and single quantum (SQ1, SQ2) components of the effective Hamiltonian. There are two single-quantum components corresponding to single-quantum coherence on the electron (S"!I"") and the nuclear spin (S""I"!), respectively." We hope that these changes make the text clearer.

Page 7/21, line 10: If n = 3, what is the corresponding mixing time? I assume it must be on the order of a few hundred nanoseconds (< 1 μs) before T_2e relaxation becomes significant. Later in the manuscript, the authors mention that 72 pulses × 5 ns = 360 ns were used. Does this imply that n = 1 corresponds to 24 pulses?

This is obviously an unclear formulation in our initial manuscript. One basic DNP block of an optimized pulse sequence consists of N pulses with a pulse length of 5 ns. The number of pulses for the different sequences is shown in Tab. S1 in the SI. The modulation time tau_m of a specific sequence is tau_m= N*5 ns. The basic DNP block is then repeated n times as shown in Fig. 1 (A) in the main text. Thus, the total contact time is t_{con} = n*tau_m. The number n is optimized experimentally and a value of n = 3 was found to perform best for both the on- and off-resonance sequence. To make an example: a sequence with N=72 pulses has then a contact time t_{con} = 3*72*5 ns = 1080 ns.

We added in the main text at the end of page 6 the following sentences:

"A basic DNP block consists of N pulses, each with a duration of $\tau_{\#}$. Thus, the modulation period is $\tau_{\$} = N\tau_{\#}$."

Page 9/21, line 9: The reported enhancement factors of 23 and 70 appear low compared to values in other pulsed DNP studies, which often exceed 300 under optimized or saturated conditions. Is this primarily due to the relatively short 2-s DNP build-up time used here? Would the authors expect enhancements above 200 if longer build-up times (>10 s) were employed?

Yes. this is correct. We used a rather short overall DNP build-up time of 6 s to reduce the measurement time for a DNP profile. The maximum of the build-up curves for the sequences can be found in the SI and is roughly 120. We did not optimize the experiments for maximum enhancement.

Figure-Related Comments

Figure 1A: Since the entire subfigure (A) is located on the left, the word “left” is redundant. The caption could be simplified by changing “top left” to “top” and “middle left” to “middle.” Thanks for mentioning this. We updated the figure caption.

Figure 2: Are subfigures (A) and (D) slices extracted from the 2D plots in (B) and (E) at a microwave amplitude of 1 (i.e., along the y-axis)? Clarification would help readers interpret the figure.

We agree that this is an unclear formulation that can lead to confusion. In subfigures (A) and (D) we show experimental DNP profiles. In subfigures (B) and (E) we show the numerically calculated transfer efficiencies using the optimized DNP pulse sequences.

We modified the caption of Fig. 2 (B) and (E): “Numerical calculation of transfer efficiency as function of the normalized microwave amplitude (25 MHz and 20 MHz, respectively) and the electron frequency offset $\Delta w/(2\pi)$ for the two sequences”

Figure 4: The labels (A/B/C) are missing from the figure panels. There is also a typographical error in the caption (“centered at 50…”).

Thank you for mentioning this. We added the label A), B) and C) in Figure 4 in the main text.

Figure 4C: Regarding the mismatch between experiment and simulation/theory: the simulations use a two-spin model. Could multispin effects be relevant, especially at large offsets? For example, two-nuclear-spin contributions may become significant (see DOI: 10.1126/sciadv.aax2743).

We agree that three-spin effects can be responsible for deviation between experiment and simulation/theory. We added an extension to the explanation and referenced the paper (DOI: 10.1126/sciadv.aax2743).

We added the text ”Another reason for the discrepancy between experimental profiles and simulated ones result from three-spin effects as observed experimentally for the SE in Ref. and shown in Fig. In the SI. This effect is not covered by our two-spin simulations.” on page 12 of the main text.

Additional Methodological Clarification

GAMMA simulations: The manuscript would benefit from a more detailed description of the spin parameters used in the GAMMA simulations. Specifically, please provide the values of T_1n, T_2n, T_1e, T_2e, and the hyperfine tensor employed.

The relaxation parameters were obtained from experimental data except for $T_{2,H}$. The GAMMA source code is included in the repository. We added Section E in the SI to describe the GAMMA simulations including all input parameters.

Reviewer: 2

Recommendation: This paper may be publishable, but major revision is needed; I would like to be invited to review any future revision.

Comments:
This manuscript describes the theoretical analysis of pulse DNP sequences (on-resonant and off-resonant) by continuous Floquet theory. This is an extension of an earlier work of this research group (PCCP 2024) using operator-based and matrix-based discrete Floquet theory. The authors use and demonstrate their approach by optimizing an on-resonant and off-resonant pulse sequence with amplitude modulated MW pulses with regard to the zero-quantum operator between electron and nuclear spin. This is interesting, since pulse DNP sequences have a large potential to increase DNP efficiency for polarizing agents with broad EPR spectra and potentially also at high magnetic fields (if the necessary pulse and frequency modulated MW power becomes technically available in the future). My main points of critic with the existing manuscript are:

The authors claim that their approach is superior to already existing approaches (as Single-Spin Vector effective Hamiltonian Theory) because it can better treat near-resonance conditions. This is important because this conditions will always appear with the limited MW power available. Unfortunately, I do not see in the paper a demonstration of this superior behavior. The authors only show comparison with numerical GAMMA spin dynamic simulations, which demonstrate that the approach is valid, but does not demonstrate that it is superior to SSV-EHT.

Thanks for pointing this out. It is not our intention to state that our numerical optimization procedure using effective Hamiltonians derived from continuous Floquet theory is superior to the SSV-EHT method. Our main message of the paper is to provide an alternative optimization method to the existing ones. However, our procedure is different from other

optimization methods because the terms in the effective Hamiltonian that are responsible for the polarization transfer enter directly into the cost function. This is only possible within the framework of continuous Floquet theory, due to the broadening of resonance conditions arising from the convolution of the discrete Fourier-series representation with a sinc function that allows a smooth transition from resonant to non-resonant contributions.

We agree with the misleading of our formulation and removed all statements claiming an improved transfer efficiency of our optimized sequences (see also next point).

Secondly, the authors have used their approach to optimize an on-resonance MW pulse and compare it with the published PLATO sequence (optimized with SSV-EHT). Their analysis of the results are somewhat confusing. They state that their new developed MW pulse is superior because it achieves the same bandwidth with somewhat reduced MW power (20%). Later-on, they state that the enhancement of their pulse sequence is 20% lower! It is not clear to me, why they did not compare with the same maximum MW power (which was available for their PLATO control experiment) and why they are not more precise in this comparison. By the way, the reader has to guess in Figure 3A which trace belongs to the new sequence and which one to the PLATO sequence; this has to be explained! I would propose, that the authors make these two points more clear in a revised version of the manuscript before publication!

Thank you for mentioning this. The legend in Fig 3A was missing and led to confusion. We added the legend to this figure. We recorded the PLATO sequence and compared it to our optimized sequence for two reasons: (1) We demonstrate the proper functioning of our experimental setup by reproducing a previously published sequence. While this may seem redundant, it confirms that our setup can generate DNP sequences with phase modulation on a 5 ns timescale. (2) Our goal with this comparison is to demonstrate that a same bandwidth can be achieved with reduced mw amplitude (20% reduction in amplitude). We believe this is an important point, as at larger magnetic fields the maximum microwave amplitude is limited, while a large bandwidth of DNP sequences is desired, particularly for broadband radicals. A reduced microwave amplitude, on the other hand, inevitably leads to a reduced enhancement as pointed out in Ref. DOI: 10.1039/d4cp01788a. Overall, this highlights the trade-off between maximum achievable bandwidth and maximum enhancement.

To clarify those points, we added the following sentences:

Page 9: We changed the sentence “The experimental comparison shows that our sequence can cover the same bandwidth with a similar enhancement value but using about 21% less microwave amplitude.” to

“The experimental comparison shows that our sequence can cover the same bandwidth by using about 20% less microwave amplitude. However, the maximum achieved enhancement is approximately 20% (60 vs. 72) lower. This illustrates the trade-off between maximum achievable bandwidth and enhancement, which becomes particularly relevant at larger magnetic fields.”

Page 14: We changed the sentence “Our optimized sequence achieves a similar enhancement value and spans the same bandwidth efficiently while requiring about 21% less microwave amplitude, demonstrating improved transfer efficiency. While the total enhancement is higher for PLATO, reaching a maximum of about 72 compared our sequences still shows a comparable enhancement of about 60 (~20% lower).” to

“Our optimized sequence uses about 20% less microwave amplitude and, therefore yields about 20% less enhancement, but achieves the same bandwidth. This result illustrates the interplay between the maximum achievable bandwidth and enhancement at a given available microwave amplitude.”

Minor points:

Despite it was already done like that in earlier publications I would favor to have the pulse amplitudes shown from +1 to -1 in one color instead of showing only the magnitude of the B1 field with two colors.

Thanks for this comment. To remain consistent with earlier publications, we retained the established graphical representation of the optimized pulse sequences. Moreover, a figure with amplitudes ranging from +1 to −1 would require more space. Therefore, we would like to keep it that way.

Please give the pulse length of the MW rectangular pi/2 pulse.

We agree with this point. The pulse length of the rectangular pi/2 pulse is 6 ns with a Rabi frequency of 41.667 MHz.

We added on page 7 the sentence: “For the on-resonance experiment a 90° pulse with a length of 6 ns and phase +y was inserted before the DNP module.”.

The authors describe that for on-resonant cases they start from the Sx vector. On the other hand, with the given MW field strength, this is not exactly correct. For off-resonant spins the starting vector will deviate. Anyway, to me it is unclear, why this first pulse is not included in the whole calculation.

Thanks for mentioning this. In the GAMMA simulations the pi/2 pulse is included into the calculations as a “real” pulse, i.e., $U=expm(-i*(H\_spin+w_1S_y)*t)$. On the other hand, in the

effective Hamiltonian calculation the magnetization is starting from the S_x vector assuming a perfect pi/2 pulse. However, the FWHM of the EPR spectrum is around 6.3 MHz. A pi/2 pulse with a length of 6 ns and a Rabi frequency of 41.667 MHz has an excitation bandwidth which is much broader than the narrow EPR line of the Ox063 trityl radical. We expect that the small fraction of off-resonant spins which are not completely aligned along the S_x vector have a negligible effect in experiments and simulations. This can also be seen by comparison of GAMMA simulations with effective Hamiltonian calculations.

We added in Fig. S6 in Section F of the SI the theoretical excitation pulse profile of the pi/2 pulse.

The authors write about tolerance of their sequence against MW field strength inhomogeneity, but I did not find how this was extracted and how such profiles were taken into account.

Thank you for the remark. The MW field strength inhomogeneity was accounted for by a power model as described in Ref. (DOI: 10.1016/j.ssnmr.2015.09.005). This was mentioned in the original paper draft on page 5 in the sentence: “A simple power distribution model was used to address microwave inhomogeneity[37], with normalized amplitude values of (1.05, 1.00, 0.95, 0.85) and corresponding weights of (0.1783, 0.3856, 0.2461, 0.1900).” We have modified this sentence in the main text to clarify how the microwave field inhomogeneity enters in the numerical optimization procedure. The new sentence is as follow:

“The microwave inhomogeneity in the optimization procedure was accounted for by using a simple power distribution model.[21,36] This is implemented in the optimization procedure via a normalized pulse amplitude vector (1.05, 1.00, 0.95, 0.85) with weights (0.1783, 0.3856, 0.2461, 0.1900) of the corresponding cost function.”

We agree that this is a very simple model. However, we choose such a model for two reasons. (1) A more complicated model for the MW field strength inhomogeneity based on approach similar to the ones proposed for the rf inhomogeneity (see Refs. https://doi.org/10.1016/j.jmr.2017.09.002 or https://doi.org/10.1002/cmr.10029) would slow down the numerical optimization drastically (2) Accurately characterizing the microwave field strength inhomogeneity present in the resonator during the measurement with experiments or simulations is not straightforward.

Please also specify the ZZ Term (Spin-alignment) in the manuscript

See the comments to the same question by reviewer 1.

The authors write about power droop during the pulse. How was this evaluated? How was the TWT non-linearity at high MW powers taken into account? Is there also a MW phase shift in the non-linear regime?

The droop of the TWT amplified mw pulses was measured with the following protocol:

The mw signal amplified by the TWT was passing a first 3-port directional coupler (narda 4015C-30, frequency range 7-12.4 GHz). Almost all of the mw signal was directed towards the resonator. -30 dB of the initial mw signal was transmitted through a second 3-port directional coupler of the same type for further attenuation of the mw irradiation before passing it through a diode (DD-20-218-5PF-3-P-M, frequency range 2-18 GHz, rise time about 5 ns). The attenuated mw signal was then measured with an oscilloscope (Rhode Schwarz RTA 4004). We added this paragraph and a figure of the droops measured in Section G of the SI.

- The TWT non-linearity is taken into account not only at high mw amplitude, but of course has the strongest influence at high mw amplitudes. The experimental description of the experiment to record the TWT non-linearity is given in Section A of the SI of the original draft. Fitting for both the dependence of Rabi frequency on digital amplitude and the dependence of digital amplitude on required Rabi frequency of the TWT non-linearity curve by polynomials of fourth order allows the mapping between the digital amplitude and the Rabi frequency. This is needed to have an accurate control over the Rabi frequency in a DNP pulse sequence. The TWT nonlinearity curve together with the resonator profile is used to compensate for the limited width of the microwave resonator mode and differences in non-linearity of the TWT during the acquisition of a DNP profile.

The mw phase shift is an important point to be considered. The current implementation of the mw bridge uses a single channel excitation. So, the “ground” phase is defined at the output of the AWG and we have digital control over it. The AWG we use is from Keysight model M8190A and operates with an internal sample clock at 10 GSa/s and a frequency of 1.28 GHz. According to the data sheet of our AWG (https://www.keysight.com/us/en/assets/7018-02903/datasheets/5990-7516.pdf) the phase noise with internal sample clock at 12 GSa/s for a carrier frequency of 1.5 GHz at an offset frequency of 1 kHz is around -100 dBc/Hz. For larger offset frequencies, the phase noise is even lower.

jz-2025-03855p.R2

Name: Peer Review Information for "Effective Hamiltonian Based DNP Sequence Optimization"

Second Round of Reviewer Comments

Reviewer: 1

Comments to the Author

I am satisfied with the response, and the paper can be published as is. I have two minor points that the authors can choose to address if they wish.

Regarding the depositing MATLAB, numerical simulations, or any other script on online depository. The authrors's response on this 'In the longer run, we will also make the implementation publicly available ....' sounds like a typical standard reply (and very much expected) from the group whenever the authors are asked to share their scripts online. In fact, I am personally not aware of much scripts shared by the authors online (raw data and results are a different story, but they are not very useful towards methodological development by other groups). Anyway, I can only encourage (repeatedly) urge the users to share their know-how in an online depository on GitHub for instance, which can be updated as frequently as the authors wish..

Secondly, I forgot to ask this during the first round of review, can the authors numerically optimise the scaling factor of the offset term in the ZQ/DQ subspace, to implement an adiabatic sequence? I would imagine that an adiabatic sequence will yield more broadband or robust performance relative to the existing one. I am aware that this might be asking too much, but I will appreciate if the authors can at least mention this possibility or prospect in the conclusion.

Reviewer: 2

Comments to the Author

The authors addressed all the questions and remarks made by both reviewers. I am happy to accept the manuscript with the suggested additions and revisions now as it

is for publication

Author's Response to Peer Review Comments:

In the following response letter, the reviews' comments are shown in **black**, and responses from the authors are shown in **red**.

Note: Panel F of Figure 2 has been revised to present the correct values of the effective Hamiltonian terms. This correction does not affect any of the conclusions of the manuscript.

Reviewer: 1

Recommendation: This paper represents a significant new contribution and should be published as is.

Comments:
I am satisfied with the response, and the paper can be published as is. I have two minor points that the authors can choose to address if they wish.

Regarding the depositing MATLAB, numerical simulations, or any other script on online depository. The authrors's response on this 'In the longer run, we will also make the implementation publicly available ....' sounds like a typical standard reply (and very much expected) from the group whenever the authors are asked to share their scripts online. In fact, I am personally not aware of much scripts shared by the authors online (raw data and results are a different story, but they are not very useful towards methodological development by other groups). Anyway, I can only encourage (repeatedly) urge the users to share their know-how in an online depository on GitHub for instance, which can be updated as frequently as the authors wish..

We are unsure where the reviewer got the notion that we do not publish code. All papers from the past two years from our group include the full scripts needed to reproduce every simulation and processing step. It is possible to find such script together with the data in public repositories such as the ETH Research Collection or Zenodo. We would like to remark that we fully support open sharing of code, but it should be provided in a form that is functional and usable by anyone. A more generalized, expanded and well-documented version of the simulation scripts used for this

publication will be released with our upcoming methodological paper on effective Hamiltonian optimization.

Secondly, I forgot to ask this during the first round of review, can the authors numerically optimise the scaling factor of the offset term in the ZQ/DQ subspace, to implement an adiabatic sequence? I would imagine that an adiabatic sequence will yield more broadband or robust performance relative to the existing one. I am aware that this might be asking too much, but I will appreciate if the authors can at least mention this possibility or prospect in the conclusion.

We thank the reviewer for this comment. This is an interesting point that was not addressed in the manuscript. In principle, the reviewer is correct that an adiabatic sequence could yield more robust control. However, implementing an optimized adiabatic sequence is not straightforward within our current optimization framework. Our approach operates in the fully interacting frame, which allows us to include the effective fields in the effective Hamiltonian. A proper implementation would require to take the effective fields out and controlling their magnitudes, but we believe that such further implementation lies beyond the scope of the present work.

We acknowledge the review suggestion by adding in the conclusion of the manuscript the following sententes:

"In principle, such sequences could also be implemented in an adiabatic fashion by sweeping through the resonance condition. This could be implemented by scaling the length and the amplitude of the pulses such that the effective flip angle remains unchanged or by sweeping the static magnetic field. Both options would generate a sweep through the resonance condition which is required for an adiabatic transfer. However, none of these options have been explored so far and we are not sure whether the complete interaction frame used in this work is the best framework to describe such adiabatic sequences."

Reviewer: 2

Recommendation: This paper represents a significant new contribution and should be published as is.

Comments:
The authors addressed all the questions and remarks made by both reviewers. I am happy to accept the manuscript with the suggested additions and revisions now as it is for publication.

We thank the reviewer for the interesting comments and suggestion addressed during the peerrevier process.
